# Evaluation of the effectiveness of the Global Medical Student Partnership program in undergraduate medical education

**DOI:** 10.36834/cmej.69339

**Published:** 2020-12-07

**Authors:** Hannah Samuels, Vanessa Rojas-Luengas, Arman Zereshkian, Shirley Deng, Jenna Moodie, Paula Veinot, Ashna Bowry, Marcus Law

**Affiliations:** 1 MD Program, Faculty of Medicine, University of Toronto, Ontario, Canada; 2 Department of Family and Community Medicine, University of Toronto, Ontario, Canada; 3 Unity Health Toronto, Ontario, Canada; 4 Toronto Rehabilitation Institute, University Health Network, Ontario, Canada

## Abstract

**Background:**

The Global Medical Student Partnership (GMSP) is a medical student-led international initiative to promote accessible global health learning. This study aims to evaluate the effectiveness of the GMSP program in meeting its learning objectives.

**Methods:**

Canadian and international medical student pairs met online monthly (January-May 2018) to discuss global health-related medical cases. Students then reviewed cases with local GMSP peers and faculty experts. A mixed-methods study was performed to evaluate whether the objectives of the program had been achieved. 26 of 32 (81.3%) students completed a questionnaire, and 13 (40.6%) also participated in one-on-one semi-structured interviews. Descriptive statistics and thematic analysis were used to analyze students’ perspectives on skill development through GMSP.

**Results:**

GMSP students agreed or strongly agreed that international collaboration and communication skills were more important to them following program participation (92.3%, 92.3% respectively). Many expressed that after GMSP, they knew more about their healthcare system, practices abroad and how to solve complex health issues (92.3%, 84.6%, 61.5% respectively). Qualitative data showed GMSP improved students’ communication and presentation skills, provided a foundation for international relationships, fostered appraisal of diverse health systems, and furthered students’ understanding of health advocacy.

**Conclusions:**

Our findings demonstrate that GMSP met its original objectives by providing students with opportunities to engage in international collaborations and to further develop their skills in advocacy, communication, and health-systems research. This program may be an important addition to medical education as it makes use of technology and peer-to-peer exchange to enable global health learning.

## Abstract (fr)

**Contexte:**

Le Global Medical Student Partnership (GMSP) est une initiative d’envergure internationale menée par des étudiants en médecine qui vise à favoriser la formation en santé mondiale. La présente étude consiste à évaluer l’efficacité du programme GMSP pour atteindre ses objectifs d’apprentissage.

**Méthodologie:**

Des paires d’étudiants en médecine canadiens et étrangers se sont rencontrés en ligne tous les mois, entre janvier et mai 2018, pour discuter de situations cliniques en santé mondiale. Après la rencontre, ces situations cliniques ont été revues par des pairs locaux du programme GMSP et des experts du corps professoral. On a effectué une étude à devis mixte pour déterminer si les objectifs du programme avaient été atteints. 26 des 32 (81,3 %) étudiants ont répondu à un questionnaire et 13 (40,6 %) ont aussi pris part à des entrevues individuelles semi-dirigées. Des statistiques descriptives et une analyse thématique ont été utilisées analyser les perceptions des étudiants sur le développement d’habiletés par le programme GMSP.

**Résultats:**

Les étudiants participant au programme GMSP étaient d’accord ou très en accord pour dire que les habiletés à la collaboration internationale et à la communication étaient plus importantes à leurs yeux après la participation au programme (92.3%, 92,3%, respectivement). Bon nombre ont affirmé qu’après le programme GMSP, ils en connaissaient plus sur leur système de soins de santé, les pratiques à l’étranger et les façons de résoudre des problèmes de santé complexes (92,3 %, 84,6 %, 61,5 % respectivement). Des données qualitatives ont montré que le programme GMSP a amélioré les aptitudes à la communication et des techniques de présentation. Elles ont servi à établir des relations à l’international, à évaluer divers systèmes de soins de santé et à mieux comprendre la promotion de la santé et à militer en faveur de celle-ci.

**Conclusions:**

Nos résultats montrent que le programme GMSP a atteint ses objectifs de départ puisqu’il a donné aux étudiants des occasions de collaboration internationale et leur a permis de développer davantage leurs habiletés en matière de défense des droits, de communication, et de recherche sur les systèmes de soins de santé. Ce programme pourrait s’avérer un important complément à la formation médicale parce qu’il utilise la technologie et des échanges pairs-pairs pour l’apprentissage des enjeux de santé mondiaux.

## Introduction

As the world becomes increasingly interconnected, it is imperative for medical students to have a sound understanding of global health in order to help address health inequities within and between countries.^1^ Global health is “an area for study, research, and practice that places a priority on improving health and achieving equity in health for all people worldwide.”^2^ Despite the need for further global health undergraduate medical education, current Canadian undergraduate medical curricula have not adequately addressed this important issue.^3^ Global health curricula remain highly variable, fragmented and lacking in experiential learning opportunities.^4^

Most global health medical education in Canada is delivered through large group lectures. The experiential learning that is available is often comprised of international elective programs limited in capacity and costly to students, thus creating barriers to accessing immersive global health experiences.^5^ Furthermore, these programs are often unidirectional, providing benefits mainly to learners from high-income countries. These benefits include gaining a greater understanding of the impact of culture, socioeconomic influences and public health on the health of communities.^5^^,^^6^ With the aim of global health being to achieve equitable health for all people, it is important that international elective programs provide equitable learning opportunities.^7^ Thus, bidirectional partnerships have become a focus in global health medical education in which students from both international institutions have the opportunity to learn from medical practice at each other’s locations.^7^

Medical learners around the world at all stages of training have been requesting more widely available and accessible global health training opportunities.^5^ The Association of Faculties of Medicine of Canada (AFMC) and the Lancet Commission on education of health professionals for the 21st century recognized the need to educate healthcare professionals in all countries for quality patient-centered care. They also identified the requirement of addressing health inequities locally and globally as a fundamental principle of professionalism in medicine and a continuation of the social contract that exists between the health system and society.^1^^,^^8^

The student-run Global Medical Student Partnership (GMSP) program arose out of the gap between accessible global health learning opportunities and bidirectional international partnerships. It is offered to all pre-clerkship medical students at the University of Toronto as an extracurricular opportunity. GMSP participants connect online with international medical students to share local medical and cultural expertise, collaborate to solve common healthcare issues such as those in the fields of women’s health, health in conflict zones and traditional healing, and promote health advocacy. Canadian GMSP students then present to peers and faculty on how their communities and their international partners’ communities tackle health and social issues and learn how they may advocate on behalf of future patients.

By using peer-to-peer knowledge exchange, GMSP aims to provide opportunities for building international relationships and fostering collaboration amongst diverse medical institutions, as well as ways to develop students’ skills in advocacy, communication and health-systems research. Importantly, due to the use of free web-based technology, this is achieved at no cost. We carried out an evaluation study to understand the effectiveness of this program in meeting the aforementioned learning objectives.

## Methods

### GMSP Program design

In 2018, 20 University of Toronto first and second-year medical students were randomly partnered with international students from medical schools in Ethiopia, Israel, Jamaica, and Saudi Arabia, recruited by international medical faculty contacts known to study investigators. Student partners were assigned cases by the GMSP organizers on global topics (naturopathic medicine, women’s health, medicine in conflict zones, medical assistance in dying, pandemics, mental health), which were then reviewed in live group sessions by faculty experts. Using guiding questions developed for each case through literature review and discussion amongst study investigators, student pairs researched topics and discussed how they were approached in each partner’s country through online platforms (Skype, WhatsApp). Each pair met five times (January to May) independently in a one-on-one session. Local University of Toronto students then presented their learnings and reflections from discussions with their international partners at monthly group debriefs. These monthly debrief sessions were facilitated by experts (i.e., physicians, Aboriginal elders, bioethicists, paramedics), and were video-recorded and disseminated to international partners. A sample case has been provided in Appendix A.

### Study design

We conducted a concurrent triangulation^9^ mixed-methods study (University of Toronto Research Ethics Board approved) to evaluate the effectiveness of the GMSP program in meeting its learning objectives from the perspectives of student participants. Using a modified Dillman Method^10^ (with its focus on follow-up) as an approach to improve response rate by busy participants,^11^ an immediate post-program questionnaire with five-point Likert scales^12^ (Supplemental Material, Appendix B), and consent form (Supplemental Material, Appendix C), were distributed. Email reminders were sent at two and four weeks.

One research team member (H.S.) obtained written consent and conducted 30-60-minute recorded Skype interviews using a semi-structured interview guide (Supplemental Material, Appendix D). Interviews were transcribed for thematic analysis.^13^

### Data analysis

We analyzed the survey data using descriptive statistics. We used NVivo 11 (QSR International, Melbourne, Australia) to organize interview data. We performed thematic analysis on the qualitative interview data following the stepwise approach of Braun and Clarke.^13^ The process began by reading the data and jotting down initial thoughts to become familiar with the data. Next, we (P.V) coded data top down (using pre-identified codes based on the interview guide) and bottom up (as new codes emerged during analysis), compared iteratively, and sorted into potential themes (H.S., V.RL., A.Z). The team reviewed the evolving themes every few interviews to verify the continued fit and to name and further define the themes,^13^ thus reducing bias and allowing for refinement of questions according to emerging information.^14^ The analysis culminated in a summary of the data.^13^

## Results

### Quantitative results

Twenty-six of 32 program participants completed the questionnaire. Eleven were international (42.3%). Participants were predominantly female (*n* = 18, 69.2%), less than 25 years (*n* = 18, 69.2%), and had previously been involved in global health initiatives (*n* = 17, 65.3%). Most had an undergraduate bachelor’s degree or postgraduate master’s degree (*n* = 20, 76.9%).

After participating in GMSP, most students agreed or strongly agreed that they knew more about their own healthcare system (*n* = 24, 92.3%) and healthcare practices abroad (*n* = 22, 84.6%). Nearly two-thirds agreed or strongly agreed that they knew more about how to solve complex health issues (*n* = 16, 61.5%). Nearly all students also agreed or strongly agreed that their ability to collaborate (*n* = 24, 92.3%) and their communication skills (*n* = 24, 92.3%) were improved. Most students agreed or strongly agreed that it is important to further develop their communication skills (*n* = 24, 92.3%), and more than half of participants became more comfortable presenting in front of groups (*n* = 15, 57.7%) as a result of participating in GMSP. By being involved in GMSP, two-thirds of participants agreed or strongly agreed that they felt more confident debating complex healthcare topics (*n* = 17, 65.4%). By participating in GMSP, three-quarters of students agreed or strongly agreed that they were more likely to volunteer in underserviced communities (*n* = 21, 80.8%) or pursue a medical elective abroad (*n* = 20, 76.9%) See Table 1.

**Table 1:** Impact of GMSP on survey respondents

Survey Question	Strongly Disagree /Disagree	Neither Agree or Disagree	Agree	Strongly Agree
Improved ability to solve complex health problems	2 (7.7)	8 (30.8)	11 (42.3)	5 (19.2)
More knowledgeable about global health practices	0 (0.0)	4 (15.4)	17 (65.4)	5 (19.2)
Improved collaboration skills	0 (0.0)	2 (7.7)	10 (38.5)	14 (53.8)
More knowledgeable about my own country’s healthcare system	0 (0.0)	2 (7.7)	16 (61.5)	8 (30.8)
Improved communication skills	0 (0.0)	2 (7.7)	15 (57.7)	9 (34.6)
More weight placed on importance of communication	0 (0.0)	2 (7.7)	15 (57.7)	9 (34.6)
Improved comfort presenting to large groups	1 (3.9)	10 (38.5)	11 (42.3)	4 (15.4)
Increased confidence debating complex topics	0 (0.0)	9 (34.6)	13 (50.0)	4 (15.4)
More likely to volunteer locally	0 (0.0)	5 (19.2)	14 (53.8)	7 (26.9)
More likely to pursue medical electives abroad	0 (0.0)	6 (23.1)	13 (50.0)	7 (26.9)

### Qualitative findings

Thirteen students (40.6%) were interviewed, 11 of whom were Canadian (84.6%). Two-thirds were female (*n* = 9, 69.2%). Themes were organized in relation to program goals, with sample quotations in Table 2.

**Table 2:** Supporting quotations GMSP participants’ direct quotations indicating improvements in all variables of interest

Theme:	Representative Quotations
GMSP improved students’ ability and confidence related to communication and presentation skills	Explaining our own opinions and our own cultural values and cultural norms to a person who doesn’t necessarily understand them was good practice in communication and collaboration. - #12 Canadian Female Year 2 [GMSP] gave me the comfort and helped me to practice sharing my opinion in a peer group and how to talk with somebody about […] a controversial issue in a respectful way. - #29 Canadian Female Year 1
GMSP provided a foundation for the development of relationships that foster open dialogue and global learning	Being able to communicate with somebody from a different culture and different system, I definitely think it really emphasizes the whole global nature of global health. We have to be able to communicate with our colleagues, we have to be able to understand each other’s systems- our pros, our cons, our strengths, our weaknesses and leverage each other’s strengths. - #33 International Male Year 4 [My partner] taught me a lot about the culture. It’s one thing to read about a place, but to really get a feel about the culture you really have to talk to the locals […] One of the things she talked about which was actually shocking was that […] you hear about the Native Americans in America. I didn’t know that there was an analogue to that in Canada. And the treatment of […] Indigenous people seems to be an issue all over the world. - #15 International Male Year 3
GMSP provided an opportunity to learn about and evaluate health systems	I learned more about the Canadian healthcare system and how healthcare in Ontario works in addition to learning about other countries, which was really cool for me. - #5 Canadian Female Year 1 I didn’t know there was a waiting gap for three months before, when people arrive in Canada, before they get access to health care. I think that I came back to Canada knowing that it’s universal healthcare coverage and this and that, but I’m learning that there are a lot of populations or gaps where people don’t get coverage, or care is available but it is years out and maybe not as accessible when people need it. - #29 Canadian Female Year 1
Students developed an understanding of components required to advocate for patients locally and globally	Often times […] our partners are not present in the room to hear the case with us so we would have to present our collective answers […] so I had to advocate on [my partner’s] behalf and also advocate on the responses he had. - #8 Canadian Male Year 1 I think we […] discussed and reflected on the diversity of experiences people can have […] If you want to do advocacy successfully, you need to do it in partnership with the person you’re advocating on behalf of […] I can’t relate if I’m not part of that group, so discussing different health challenges that different populations experience, knowing and being aware of that will be how I devote my advocacy efforts. - #5 Canadian Female Year 1

### GMSP improved students’ ability and confidence related to communication and presentation skills

Many students identified that GMSP enabled them to further build their communication skills and reported that the interactions with their partners taught them cultural awareness and ways to concisely and coherently explain new concepts. Furthermore, several students commented on becoming more comfortable presenting their ideas and advocating for their international partners in front of a group.

### GMSP provided a foundation for the development of relationships that foster open dialogue and global learning

GMSP students reported their relationships with their assigned partners to be one of the most valuable experiences within the program. Over time interactions became less formal, and more open and relaxed. They described being humbled when their assumptions about healthcare systems abroad were proven to be wrong.

Students stated that they would feel comfortable visiting their GMSP partners during future electives in their respective countries. Several pairs remained connected through social media.

### GMSP provided an opportunity to learn about and evaluate health systems

GMSP cases prompted students to explore services available in their local communities, to critically evaluate healthcare systems, and to learn more about how people in other countries experience similar health issues.

Many students found that the panel discussions with experts formed a large component of their learning. Many local students found that accessing policy statements or Canadian regulations on specific health topics was not as straightforward as originally thought. Several students discovered that their assumptions regarding Canadian laws and provision of healthcare services were inaccurate.

### Students developed an understanding of components required to advocate for patients locally and globally

While most participants did not view GMSP as an explicit advocacy program, several mentioned that it provided exposure to researching a topic, forming an opinion, conveying an argument, and communicating with people who have different ideas, which are all important aspects of advocacy.

Other students found themselves acting as advocates for their partners by sharing their partners’ views (in their absence) at the group debrief sessions. This further translated into developing a deeper understanding of how to advocate for future patients.

## Discussion

Evidence suggests that global health education is critical in preparing healthcare professionals to meet the health needs of our diverse populations. It is also thought that cross-cultural medical education can lead to better physician-patient communication, help eliminate racial disparities and improve cultural sensitivity, resulting in increased patient satisfaction, adherence, and better health outcomes.^15^ Although the need for global health curricular content is clear, the limited research conducted mostly describes an insufficient response by medical schools to the increased student demand for global health content.^16^ Currently, most teaching consists of large group lectures, modules, and/or international elective opportunities that are often non-uniformly structured with poor faculty supervision.^5^^,^^6^ Importantly, despite the clear need for international collaboration, there previously existed no program similar to GMSP at the undergraduate medical level.

Our data suggest that GMSP is effective in enhancing skills related to communication, collaboration, health systems research, person-centred care, and health advocacy, with respect to global and local health. It also provides knowledge and skill development related to the seven CanMEDS roles, all qualities deemed necessary to be an effective physician.^17^ Our data align with the various professional bodies for medical training (i.e., the Global Health Education Consortium (GHEC) and the AFMC of Canada’s Global Health Resource Group (GHRG) which have standards requiring that cross-cultural information be taught in undergraduate medical education.^16^ GMSP offers bidirectional benefit through peer-to-peer knowledge exchange, allowing students to gain first-hand experience in culturally-specific topics without having to travel, thereby eliminating financial and logistical barriers to obtaining international medical experience. This method of learning promotes application of knowledge to tackle real-world problems.^18^ The communication skills gained are important in building culturally safe medical practices and empowering students to advocate for positive change. The collaboration not only strengthens connections between current medical students and faculty in participating schools, but also provides an increased potential for international partnerships as future physicians.

We have identified several study limitations. While many international students responded to the survey, only two of our interview participants were international. This is likely due to the different time zones and exam schedules of international medical students, as well as their at-times limited internet access, making it challenging to arrange interviews. Additionally, greater than 60% of GMSP students reported prior global health experience, potentially biasing our sample. Also, since the research team was comprised primarily of the developers of the GMSP program, confirmation bias was a risk. This bias was limited by assigning one researcher to conduct all of the interviews and using triangulation to ensure that questions and interpretations of the data were appropriate.

## Conclusion

This study demonstrates the effectiveness of the GMSP program in meeting its learning objectives. The GMSP program is an important addition to extra-curricular medical education as it provides hands-on global health training, currently lacking in medical curricula. This is achieved without necessitating travel and affords benefits to students by improving their CanMEDS skills, as outlined in the CFMS 2015 National Consensus on core curriculum global health competencies aimed to prepare medical students to respond to the diversity of individuals in Canada and abroad.^3^

It is our aim to continue expanding the current GMSP program as an extracurricular opportunity incorporating additional global learning sites. In doing so, we hope that this program will improve patient and population care through shared effort and advocacy, and promote a future of sensitive, informed, and competent physicians able to effectively confront existing and prospective health challenges and inequities.

## Appendix A

### Cases - Brief description of approach

Student participants are provided a newspaper or recent journal article that is relevant to the explored topic (e.g., Medical Assistance in Dying as shown below). Student pairs (one Canadian student and one international student) then work through guiding discussion questions to help them become acquainted with the topic. Questions typically focus on exploring the topic, understanding what jurisdictional difference exist in how healthcare is delivered, and different societal perceptions around the topic. The students then submit their answers and discussions to GMSP organizers. Canadian students debrief at the end of the month at an in-person session with a content expert (in the example below, a palliative care specialist with a focus in administering MAiD) and discuss these questions, as well as other interesting observations that may have arisen during their student pair interactions with their international partners. The debrief session is recorded and provided to students abroad.

### Sample Case: Medical Assistance in Dying (MAiD)

1. Case components:
  1. Students review a Canadian newspaper article on MAiD
  2. Discussion questions*
    - In what countries is Medical Assistance in Dying legal? What is your country’s stance on MAiD?
    - What eligibility criteria must be met for a person to access MAiD?
    - What impact does MaiD have on the families of patients involved?
    - What impact does MAiD have on the doctors involved?
    - How does society view MAiD in your country?
    - What options does a doctor have who does not want to assist a patient in dying?
    - What concerns have been expressed over the legalization MAiD has on society’s view on death and dying?

*Only a subset of questions was provided in this sample to illustrate the type of questions GMSP partners discussed

## References

[1] Frenk J, Chen L, Bhutta ZA, et al Health professionals for a new century: transforming education to strengthen health systems in an interdependent world. The Lancet. 2010;376(9756):1923-58. 10.1016/S0140-6736(10)61854-5 21112623

[2] Koplan JP, Bond TC, Merson MH, et al Towards a common definition of global health. The Lancet. 2009;373(9679):1993-5. 10.1016/S0140-6736(09)60332-9 PMC9905260 19493564

[3] Gao G, Kherani I, Halpine M, et al Global health core competencies in undergraduate medical education, 2015 Available at: https://www.cfms.org/files/position-papers/2015 Global Health Core Competencies.pdf [Accessed June 16, 2020].

[4] Arthur M.A, Battat R, Brewer T.F Teaching the basics: core competencies in global health. Infect Dis Clin North Am. 2011;25(2), 347-58. 10.1016/j.idc.2011.02.013 21628050 PMC7135705

[5] Izadnegahdar R, Correia S, Ohata B, et al Global health in Canadian medical education: current practices and opportunities. Acad Med. 2008;83(2):192-8. 10.1097/ACM.0b013e31816095cd 18303368

[6] Asgary R, Junck E New trends of short-term humanitarian medical volunteerism: professional and ethical considerations. J Med Ethics. 2012;39(10): 625-31. 10.1136/medethics-2011-100488 23236086

[7] Arora G, Russ C, Batra M, et al bidirectional exchange in global health: moving toward true global health partnership. Am. J. Trop. Med. 2017;97(1):6- 10.4269/ajtmh.16-0982 PMC5508910 28719333

[8] ABIM Foundation. American Board of Internal Medicine; ACP-ASIM Foundation. American College of Physicians-American Society of Internal Medicine; European Federation of Internal Medicine. Medical professionalism in the new millennium: a physician charter. Ann Intern Med. 2002;136(3), 243-6. 10.7326/0003-4819-136-3-200202050-00012 11827500

[9] Creswell JW, Plano Clark VL, Gutmann ML, Hanson WE Advanced mixed methods research designs In: Tashakkori A, Teddlie C, editors. Handbook of mixed methods in social and behavioral research. SAGE; Thousand Oaks, CA: 2003; 209-240.

[10] Dillman DA *Mail and internet surveys: the tailored design method* Hoboken, NJ: Wiley; 2007.

[11] Thorpe C, Ryan B, McLean SL, et al How to obtain excellent response rates when surveying physicians. Fam Pract 2009;26: 65-68. 10.1093/fampra/cmn097 19074758

[12] Likert R A technique for the measurement of attitudes. Arch Psych. 1932;22(140):1-55.

[13] Braun V, Clarke V Using thematic analysis in psychology. Qual Res Psychol. 2006;3:77-101. 10.1191/1478088706qp063oa

[14] Marshall MN Sampling for qualitative research. Fam Pract. 1996;13(6):522-6. 10.1093/fampra/13.6.522 9023528

[15] Smedley BD, Stith AY, Nelson AR, editors Unequal treatment: confronting racial and ethnic disparities in health care [Internet]. Washington, DC: National Academies Press (US); 2003 Chapter 6, Interventions: cross-cultural education in the health professions. Available from: https://www.ncbi.nlm.nih.gov/books/NBK220364 [Accessed May 24, 2020]. 25032386

[16] Arthur MA, Battat R, Brewer TF Teaching the basics: core competencies in global health. Infect Dis Clin North Am. 2011;25(2):347-58. 10.1016/j.idc.2011.02.013 21628050 PMC7135705

[17] Frank JR, Snell L, Sherbino J, eds CanMEDS 2015 Physician Competency Framework. Ottawa: Royal College of Physicians and Surgeons of Canada, 2015 Available at http://www.royalcollege.ca/rcsite/documents/canmeds/canmeds-full-framework-e.pdf [Accessed June 16, 2020].

[18] Unicef. What is knowledge exchange? https://www.unicef.org/knowledge-exchange/ [Accessed June 16, 2020].

